# Clinical risk factors associated with poor prognosis in gastric cancer patients with peritoneal metastasis

**DOI:** 10.1097/MD.0000000000047944

**Published:** 2026-03-20

**Authors:** Airu Su, Ran Guo, Chao Wu

**Affiliations:** a Department of Oncology I, The Second Hospital of Hebei Medical University, Shijiazhuang, Hebei, China; b Department of General Surgery III, The Second Hospital of Hebei Medical University, Shijiazhuang, Hebei, China.

**Keywords:** ascites grade, gastric cancer, peritoneal implantation stage, peritoneal metastasis, prognosis, systemic chemotherapy

## Abstract

This retrospective study aimed to identify clinical risk factors associated with poor prognosis in elderly patients with gastric cancer with peritoneal metastasis. A total of 150 patients aged ≥65 years, treated at our institution between January 2019 and December 2023, were included. Eligible patients had histopathologically confirmed gastric adenocarcinoma with peritoneal metastasis verified by intraoperative findings, cytology, or imaging. Clinical, pathological, and laboratory data – including peritoneal implantation stage, ascites grade, Eastern Cooperative Oncology Group performance status, serum albumin, hemoglobin, neutrophil-to-lymphocyte ratio, and treatment modalities – were collected and analyzed. Survival outcomes were evaluated using Kaplan–Meier and Cox proportional-hazards regression analyses. The median overall survival was 8.3 months. Univariate and multivariate analyses identified advanced peritoneal implantation stage (P2–P3), higher ascites grade (moderate–massive), Eastern Cooperative Oncology Group ≥2, hypoalbuminemia (<35 g/L), and elevated neutrophil-to-lymphocyte ratio (≥3) as independent predictors of poor overall survival (*P* < .05). By contrast, systemic chemotherapy and palliative surgery were independently associated with improved survival. These findings indicate that both tumor burden–related and host condition–related parameters significantly affect prognosis. Comprehensive assessment incorporating peritoneal stage, ascites grade, functional status, and inflammatory markers may facilitate individualized therapeutic strategies and prognostic stratification in elderly patients with gastric cancer with peritoneal metastasis.

## 1. Introduction

Gastric cancer (GC) remains a leading cause of cancer mortality worldwide, and peritoneal metastasis (PM) is the predominant pattern of distant spread that confers the poorest prognosis. Contemporary population-based and clinical series indicate that peritoneal involvement is present at diagnosis or emerges as the first site of failure in a substantial proportion of patients, with median overall survival (OS) typically under 1 year despite modern systemic therapy. These observations have been reaffirmed in recent analyses that quantify the negative survival impact of peritoneal metastases relative to other metastatic sites.^[1,2]^ Within the spectrum of peritoneal disease, the magnitude and distribution of tumor burden are critical to risk stratification. Staging frameworks aligned with the Peritoneal Cancer Index (PCI) or analogous clinical categories consistently show a monotonic relationship between higher peritoneal burden and inferior outcomes, and they increasingly guide selection for intraperitoneal strategies or cytoreductive approaches in research settings.^[3,4]^ Ascites, a frequent correlate of extensive serosal dissemination and lymphatic obstruction, provides complementary, readily ascertainable information on the intraperitoneal disease environment. Recent studies and syntheses demonstrate that the presence and, more importantly, the severity of malignant ascites are associated with shorter OS and may signal relative resistance to systemic therapy.^[5]^

Host-related factors further modify prognosis by reflecting physiological reserve and systemic response to cancer. Performance status remains a robust, independent predictor of survival across advanced GC populations. In parallel, inflammation-based indices – particularly the neutrophil-to-lymphocyte ratio (NLR) – capture the interplay between tumor-driven inflammation and host immunity. Multiple meta-analyses and aggregated datasets published in the past 3 years show that elevated NLR is associated with shorter OS and progression-free survival in GC across therapeutic contexts, including cytotoxic chemotherapy and immunotherapy.^[5]^ Nutritional markers such as serum albumin – often reduced in the setting of cachexia, systemic inflammation, and protein losses into ascitic fluid – provide additional, independent discrimination of risk in patients with peritoneal dissemination.^[6]^ Therapeutically, first-line systemic regimens incorporating fluoropyrimidine-platinum backbones with biomarker-directed agents (e.g., anti-human epidermal growth factor receptor 2, anti-programmed cell death protein 1) constitute the standard of care; however, outcomes for peritoneal metastatic disease remain inferior to those for other metastatic patterns. Recent overviews report median OS in the range of approximately 6 to 12 months with systemic therapy, whereas investigational intraperitoneal modalities and selected cytoreductive surgery (CRS) with hyperthermic intraperitoneal chemotherapy yield heterogeneous results and are not universally recommended outside trials. Patient selection frequently hinges on peritoneal disease burden and functional status, underscoring the need for granular, clinically deployable prognostic tools.^[7,8]^

The present study focuses on elderly patients with gastric cancer with peritoneal metastasis (GCPM) and evaluates the association of peritoneal implantation stage and ascites grade, integrated with Eastern Cooperative Oncology Group (ECOG) performance status, albumin, and NLR, with OS, while accounting for antitumor interventions (systemic chemotherapy and palliative surgery). By emphasizing clinically ascertainable measures of intraperitoneal disease extent and host status, our analysis aims to refine risk stratification and to inform individualized treatment selection in a population with marginal therapeutic latitude.

## 2. Methods

### 2.1. Study design

This study was approved by the Ethics Committee of The Second Hospital of Hebei Medical University. This retrospective study included patients diagnosed with GCPM who were treated at our institution between January 2019 and December 2023. Eligible patients were required to meet the following criteria: histopathologically confirmed primary gastric adenocarcinoma with evidence of PM identified by intraoperative findings, cytological examination of ascitic fluid, or imaging modalities such as contrast-enhanced computed tomography (CT) or positron emission tomography–CT; age 18 years or older at the time of diagnosis; availability of complete clinical, pathological, and follow-up data, including demographic information, tumor staging, treatment modalities, and survival outcomes; an ECOG performance status score of 0 to 2 was required for study inclusion; patients with ECOG ≥ 3 were excluded; and a minimum follow-up duration of at least 6 months or until death. Patients were excluded if they had synchronous or metachronous malignancies other than GC; severe comorbid conditions such as advanced cardiovascular, hepatic, renal, or hematologic disease that could independently affect prognosis; a history of antitumor therapy (chemotherapy, radiotherapy, or immunotherapy) for other malignancies prior to the diagnosis of GCPM; or peritoneal carcinomatosis originating from non-gastric primary tumors, including ovarian, colorectal, or pancreatic cancer. The study was conducted in accordance with the ethical principles of the Declaration of Helsinki and was approved by the institutional medical ethics committee. Written informed consent was obtained from all living participants or from the legal guardians or next of kin of deceased patients prior to data collection and analysis. Because this was a retrospective study, a formal prospective sample size calculation was not performed. However, the adequacy of the sample size for multivariate Cox regression was assessed based on the events-per-variable principle. The number of observed events exceeded the recommended minimum of 10 events per variable included in the multivariate model, suggesting that the sample size was sufficient for stable estimation of hazard ratios.

### 2.2. Assessment of peritoneal implantation stage and ascites grade

#### 2.2.1. Peritoneal implantation stage

The extent of peritoneal dissemination was evaluated according to the Japanese Classification of Gastric Carcinoma, 15th edition, which categorizes PM into 3 stages based on intraoperative and/or imaging findings: P1, limited PM confined to the adjacent peritoneum of the stomach, including the greater or lesser omentum; P2, several scattered peritoneal implants involving the distant peritoneal surfaces, such as the mesentery or pelvic peritoneum, but not diffusely spread; and P3, extensive peritoneal dissemination with diffuse or confluent implants throughout the abdominal cavity, including the diaphragm, small bowel serosa, or paracolic gutters.

Peritoneal staging was primarily determined by intraoperative exploration, laparoscopic assessment, or contrast-enhanced CT, depending on clinical feasibility. In cases where surgical evaluation was not performed, CT or positron emission tomography–CT findings suggestive of nodular or irregular peritoneal thickening, omental caking, and mesenteric infiltration were used to estimate the extent of peritoneal spread. For statistical analysis, patients were stratified into 2 groups: limited PM (P1) and advanced PM (P2–P3).

#### 2.2.2. Ascites grade

The severity of ascites was assessed based on abdominal imaging (CT or ultrasonography) and clinical findings at the time of diagnosis. Ascites was graded semiquantitatively according to the Japanese Research Society for Gastric Cancer criteria and previous radiological studies as follows: grade 0 (none), no detectable ascites on imaging; grade 1 (mild), small amount of localized ascites, typically confined to the pelvic cavity or subhepatic space; grade 2 (moderate), moderate amount of ascitic fluid distributed in multiple abdominal compartments but without generalized distention; and grade 3 (massive), large volume of ascites causing diffuse abdominal distension or bowel loop separation, with evidence of free fluid across the peritoneal cavity.

When available, quantitative assessment was corroborated by ultrasound volumetry or CT attenuation analysis. For analytic purposes, patients were grouped into 2 categories: none–mild ascites (grades 0–1) and moderate–massive ascites (grades 2–3). Imaging findings were independently reviewed by 2 experienced clinicians. Although a formal double-blind assessment was not feasible due to the retrospective design, discrepancies were resolved by consensus discussion to minimize subjective bias.

### 2.3. Data collection

Clinical data of all eligible patients were retrospectively retrieved from the electronic medical record system of our institution. Data collection was performed by 2 independent researchers using a standardized case report form to ensure accuracy and consistency. The following variables were extracted: demographic characteristics: age, sex, and body mass index; clinical and performance data: ECOG performance status, presence of comorbidities (hypertension, diabetes mellitus, cardiovascular disease, etc), and clinical symptoms at diagnosis; tumor-related parameters: primary tumor location, histological differentiation, peritoneal implantation stage, ascites grade, and presence of liver metastasis or other distant metastatic lesions; laboratory indicators: serum albumin, hemoglobin, total lymphocyte count, NLR, platelet-to-lymphocyte ratio, and tumor markers including carcinoembryonic antigen (CEA) and carbohydrate antigen 19-9 (CA19-9); treatment information: receipt of systemic chemotherapy (including regimen and number of cycles), administration of hyperthermic intraperitoneal chemotherapy if available, palliative surgery (gastrectomy or bypass procedures), and supportive care measures; and prognostic outcomes: OS and clinical status at last follow-up. All data were verified by cross-checking operative notes, imaging reports, and laboratory records. Any discrepancies were resolved by consensus between the 2 investigators. Missing values of key prognostic indicators exceeding 10% were excluded from analysis to minimize bias.

### 2.4. Statistical analysis

All statistical analyses were performed using IBM SPSS Statistics for Windows, version 28.0 (IBM Corp., Armonk) and R software, version 4.3.2 (R Foundation for Statistical Computing, Vienna, Austria). Continuous variables were tested for normality using the Kolmogorov–Smirnov test. Normally distributed data were expressed as mean ± standard deviation, whereas non-normally distributed variables were presented as median with interquartile range. Categorical variables were expressed as frequency and percentage (%). Between-group comparisons for continuous variables were performed using the independent-samples *t* test or Mann–Whitney *U* test, depending on data distribution. Categorical variables were compared using the chi-square test or Fisher exact test when appropriate. OS was defined as the time from diagnosis of PM to death from any cause or last follow-up. The Kaplan–Meier method was used to generate survival curves, and differences between groups were assessed using the log-rank test. To identify prognostic factors associated with OS, univariate Cox proportional-hazards regression analysis was first conducted to estimate hazard ratios and 95% confidence intervals for each variable. Variables with *P* < .05 in univariate analysis were subsequently included in a multivariate Cox regression model to determine independent predictors of survival. The backward stepwise likelihood ratio method was applied to optimize model fit. All statistical tests were two-sided, and a *P*-value < .05 was considered statistically significant.

## 3. Results

### 3.1. Baseline characteristics of the patients

A total of 150 elderly patients (≥65 years) diagnosed with GCPM who met the inclusion criteria were included in this study. The cohort consisted of 92 males (61.3%) and 58 females (38.7%), with a median age of 71 years (range, 65–86 years). The majority of patients (60.7%) had an ECOG performance status of 0 to 1, while 39.3% presented with an ECOG score ≥2 at the time of diagnosis. Regarding disease burden, 91 patients (60.7%) had advanced peritoneal implantation stage (P2–P3), and 87 patients (58.0%) exhibited moderate-to-massive ascites. Liver metastasis was observed in 77 cases (51.3%). Histologically, poorly differentiated adenocarcinoma was the predominant type (70.7%). The mean serum albumin level was 34.1 ± 4.8 g/L, with 56.0% of patients presenting hypoalbuminemia (<35 g/L). The median NLR was 3.2 (interquartile range: 2.4–4.8), and hemoglobin averaged 107.6 ± 18.9 g/L, with 52.0% of patients classified as anemic (<110 g/L). Elevated tumor markers were frequent, with CEA ≥ 5 ng/mL in 50.7% and CA19-9 ≥ 37 U/mL in 55.3% of patients. Regarding treatment modalities, systemic chemotherapy was administered in 89 patients (59.3%), and palliative surgery was performed in 47 patients (31.3%), while 61 patients (40.7%) received best supportive care alone. The median follow-up duration was 10.8 months (range, 2.3–34.6 months), and the median OS for the entire cohort was 8.3 months (Table 1).

**Table 1:** Univariate analysis of prognostic factors for overall survival in elderly patients with gastric cancer with peritoneal metastasis.

Variable	Category	n	Median OS (mo)	Hazard ratio (HR)	95% CI	*P*-value
Sex	Male/female	92/58	8.2/8.9	1.08	0.77–1.52	.654
Age (yr)	65–74/≥75	86/64	8.9/7.4	1.19	0.84–1.69	.329
BMI (kg/m^2^)	<18.5/≥18.5	41/109	7.1/8.8	1.33	0.93–1.92	.118
ECOG performance status	0–1/≥2	79/71	10.6/6.3	1.95	1.35–2.81	<.001
Primary tumor location	Upper/middle/lower	48/54/48	8.7/8.3/7.9	–	–	.584
Histological differentiation	Well–moderate/poor	44/106	9.8/7.5	1.27	0.87–1.86	.220
Peritoneal implantation stage (PCI)	P1/P2–P3	59/91	11.3/6.2	2.14	1.46–3.13	<.001
Ascites grade	None–mild/moderate–massive	63/87	10.7/6.1	1.96	1.34–2.85	.001
Liver metastasis	No/yes	73/77	9.8/7.1	1.51	1.06–2.15	.023
Serum albumin (g/L)	≥35/<35	66/84	10.4/6.2	1.82	1.26–2.64	.002
Hemoglobin (g/L)	≥110/<110	72/78	9.9/7.0	1.47	1.03–2.11	.034
NLR	<3/≥3	64/86	10.7/6.3	1.89	1.31–2.72	.001
CEA (ng/mL)	<5/≥5	74/76	9.8/7.2	1.45	1.01–2.09	.046
CA19-9 (U/mL)	<37/≥37	67/83	10.2/6.8	1.56	1.09–2.25	.016
Systemic chemotherapy	Yes/no	89/61	10.9/5.9	0.51	0.36–0.73	<.001
Palliative surgery	Yes/no	47/103	11.3/7.1	0.63	0.43–0.92	.017

BMI = body mass index, CA19-9 = carbohydrate antigen 19-9, CEA = carcinoembryonic antigen, CI = confidence interval, ECOG = Eastern Cooperative Oncology Group, NLR = neutrophil-to-lymphocyte ratio, OS = overall survival, PCI = Peritoneal Cancer Index.

### 3.2. Univariate analysis of prognostic factors

Univariate Cox proportional-hazards regression analysis was conducted to identify potential prognostic factors associated with OS in elderly patients (≥65 years) with GCPM. Clinical, pathological, and laboratory variables were evaluated, including demographic characteristics (age, sex, and body mass index), functional status (ECOG performance score), tumor features (primary tumor location, histological differentiation, and peritoneal implantation stage), disease burden (ascites grade and liver metastasis), nutritional and inflammatory indicators (albumin, hemoglobin, and NLR), tumor markers (CEA and CA19-9), and treatment modalities (systemic chemotherapy and palliative surgery). The results showed that advanced peritoneal implantation stage (P2–P3), high ascites grade (moderate–massive), ECOG performance score ≥2, hypoalbuminemia (<35 g/L), elevated NLR (≥3), and increased tumor marker levels (CEA ≥ 5 ng/mL or CA19-9 ≥ 37 U/mL) were significantly associated with poorer OS (*P* < .05). Conversely, patients receiving systemic chemotherapy or palliative surgery demonstrated significantly prolonged survival (Table 1).

### 3.3. Multivariate analysis of prognostic factors

All variables with *P* < .05 in univariate analysis were entered into a multivariate Cox proportional-hazards model to determine independent predictors of OS in elderly patients with GCPM. The model incorporated ECOG performance status, peritoneal implantation stage, ascites grade, serum albumin, hemoglobin, NLR, CEA, CA19-9, systemic chemotherapy, and palliative surgery. After adjustment for confounders, peritoneal implantation stage (P2–P3), ascites grade (moderate–massive), ECOG ≥ 2, hypoalbuminemia (<35 g/L), and elevated NLR (≥3) remained independent risk factors for poor OS (*P* < .05). By contrast, systemic chemotherapy and palliative surgery were independent protective factors associated with prolonged survival (Table 2).

**Table 2:** Multivariate Cox regression analysis of prognostic factors for overall survival in elderly patients with gastric cancer with peritoneal metastasis.

Variable	Category	n	Median OS (mo)	Wald χ^2^	Adjusted HR (95% CI)	*P*-value
ECOG performance status	0–1/≥2	79/71	10.6/6.3	6.30	1.68 (1.12–2.53)	.012
Peritoneal implantation stage (PCI)	P1/P2–P3	59/91	11.3/6.2	10.53	1.97 (1.31–2.97)	.001
Ascites grade	None–mild/moderate–massive	63/87	10.7/6.1	6.83	1.74 (1.15–2.63)	.009
Serum albumin (g/L)	≥35/<35	66/84	10.4/6.2	5.03	1.59 (1.06–2.39)	.025
Hemoglobin (g/L)	≥110/<110	72/78	9.9/7.0	1.09	1.23 (0.83–1.81)	.296
NLR	<3/≥3	64/86	10.7/6.3	4.38	1.52 (1.03–2.25)	.036
CEA (ng/mL)	<5/≥5	74/76	9.8/7.2	1.00	1.21 (0.83–1.78)	.317
CA19-9 (U/mL)	<37/≥37	67/83	10.2/6.8	1.58	1.26 (0.88–1.82)	.208
Systemic chemotherapy	No/yes	61/89	5.9/10.9	8.30	0.56 (0.37–0.83)	.004
Palliative surgery	No/yes	103/47	7.1/11.3	4.27	0.67 (0.46–0.98)	.039

CA19-9 = carbohydrate antigen 19-9, CEA = carcinoembryonic antigen, CI = confidence interval, ECOG = Eastern Cooperative Oncology Group, HR = hazard ratio, NLR = neutrophil-to-lymphocyte ratio, OS = overall survival, PCI = Peritoneal Cancer Index.

### 3.4. Kaplan–Meier survival analysis for key prognostic factors in elderly patients with GCPM

Kaplan–Meier survival analysis was performed to further evaluate the prognostic significance of key variables identified in the multivariate Cox regression model, including peritoneal implantation stage, ascites grade, ECOG performance status, serum albumin, NLR, systemic chemotherapy, and palliative surgery. OS was defined as the interval from the date of diagnosis of PM to the date of death or last follow-up. The survival curves were compared using the log-rank test, with *P* < .05 considered statistically significant. Patients with advanced peritoneal implantation stage (P2–P3) demonstrated markedly shorter OS compared with those with limited peritoneal involvement (P1). Similarly, those with moderate-to-massive ascites had significantly poorer survival outcomes than those with mild or no ascites. In addition, poor performance status (ECOG ≥ 2), hypoalbuminemia (<35 g/L), and elevated NLR (≥3) were associated with reduced survival. By contrast, patients who received systemic chemotherapy or underwent palliative surgery achieved notably prolonged survival compared with their counterparts who received supportive care only (Table 3).

**Table 3:** Kaplan–Meier survival analysis for key prognostic factors in elderly patients with gastric cancer with peritoneal metastasis.

Variable	Category	n	Median OS (mo)	1-yr OS (%)	3-yr OS (%)	Log-rank χ^2^	*P*-value
Peritoneal implantation stage (PCI)	P1/P2–P3	59/91	11.3/6.2	46.1/21.4	18.6/7.2	15.42	<.001
Ascites grade	None–mild/moderate–massive	63/87	10.7/6.1	44.8/22.3	17.9/8.5	12.76	<.001
ECOG performance status	0–1/≥2	79/71	10.6/6.3	49.3/25.4	21.7/10.1	10.82	.001
Serum albumin (g/L)	≥35/<35	66/84	10.4/6.2	47.9/23.5	19.8/8.6	9.57	.002
NLR	<3/≥3	64/86	10.7/6.3	48.5/26.1	20.5/9.8	8.96	.003
Systemic chemotherapy	Yes/no	89/61	10.9/5.9	53.7/18.4	23.9/6.7	14.73	<.001
Palliative surgery	Yes/no	47/103	11.3/7.1	51.1/24.8	21.4/9.2	7.94	.005

ECOG = Eastern Cooperative Oncology Group, NLR = neutrophil-to-lymphocyte ratio, OS = overall survival, PCI = Peritoneal Cancer Index.

## 4. Discussion

In this retrospective cohort of elderly patients with GCPM, several clinical and biological variables showed independent associations with OS. Advanced peritoneal implantation stage (P2–P3), higher ascites grade (moderate–massive), poor performance status (ECOG ≥ 2), hypoalbuminemia, and elevated NLR (≥3) were independently associated with increased mortality risk, whereas receipt of systemic chemotherapy and palliative surgery was associated with reduced mortality. These findings support a framework in which intraperitoneal tumor burden (peritoneal implantation stage and ascites severity) and host condition (functional status, nutritional reserve, systemic inflammation) are the principal drivers of prognosis in GCPM. Prior literature indicates that GCPM carries a short median OS under systemic therapy, typically within 7 to 15 months, which is consistent with the survival metrics observed in our cohort and underscores the clinical relevance of precise risk stratification for this population.^[9,10]^ Peritoneal disease extent was the strongest disease-specific determinant of survival. Patients classified as P2 to P3 experienced significantly inferior OS relative to those with limited peritoneal involvement (P1). This gradient mirrors the prognostic behavior of the PCI and comparable staging systems in GCPM, where higher peritoneal burden correlates with worse outcomes. Contemporary reviews emphasize that accurate quantification of peritoneal spread is central to disease stratification and therapeutic planning, aligning with our observation that peritoneal stage retains significance after multivariable adjustment.^[11]^

Ascites grade demonstrated an independent relationship with OS after controlling for peritoneal stage and other covariates. Ascites likely captures composite information on peritoneal tumor load, serosal irritation, lymphatic obstruction, and cancer-related cachexia. Recent evidence indicates that the mere presence of malignant ascites – and particularly higher grades – portends inferior survival across advanced gastrointestinal cancers and specifically in metastatic GC, which is concordant with the direction and magnitude of effect we observed. From a practical standpoint, grading ascites on routine cross-sectional imaging yields reproducible risk information without additional invasive procedures, supporting its use in routine assessment.^[12,13]^ Among host-related factors, ECOG performance status remained a robust independent predictor, reflecting global physiological reserve and tolerance for therapy. Hypoalbuminemia was associated with increased mortality, likely representing malnutrition, systemic inflammation, and disease burden. Elevated NLR (≥3) retained significance in the adjusted model, consistent with a growing body of evidence linking systemic inflammation with adverse outcomes in GC. Inflammation-based indices are increasingly recognized as pragmatic biomarkers with modest cost and broad availability; our results support incorporating NLR into routine prognostic appraisal for GCPM.^[14,15]^

Therapy-related variables showed associations with improved OS. Receipt of systemic chemotherapy conferred a survival advantage even after adjustment, consistent with contemporary benchmarks for metastatic GC under modern regimens. Similarly, palliative surgery was associated with improved survival in selected patients, in line with a recent meta-analysis suggesting a role for palliative gastrectomy in carefully chosen cases. The observed associations should be interpreted as adjusted correlations rather than proof of causal benefit; nevertheless, they reinforce the notion that active oncologic management may yield survival gains for fit elderly patients with GCPM, provided appropriate selection and goals of care.

Our findings are directionally aligned with recent reports emphasizing the prognostic impact of peritoneal disease burden and ascites in GCPM. A 2023 state-of-the-art review underscored that PM represents the dominant pattern of lethal dissemination in GC and that greater peritoneal involvement correlates with shorter survival; our multivariable results corroborate this gradient using a clinically implementable peritoneal implantation stage.^[16]^ Recent summaries of diagnostic and therapeutic strategies in 2025 reiterated the centrality of PCI-based stratification for patient selection, consistent with our finding that advanced peritoneal stage (as a surrogate for high PCI) remained independently prognostic. Ascites has reemerged as a clinically salient variable. A 2024 narrative synthesis and a 2025 meta-analysis reported that the presence and higher grades of malignant ascites are associated with significantly worse OS in metastatic GC, echoing our independent association between higher ascites grade and mortality. These data collectively support including standardized ascites grading in baseline risk assessment and follow-up.^[17]^

Inflammation-based markers have also gained traction. A 2024 meta-analysis highlighted NLR and platelet-to-lymphocyte ratio as adverse prognostic indicators in advanced GC, and a 2024 study further linked high NLR with inferior outcomes, reinforcing our adjusted association of NLR ≥ 3 with mortality. While specific cutoffs vary across cohorts, our results endorse NLR as a pragmatic, low-cost stratifier in GCPM.^[18]^ Finally, our adjusted associations for systemic chemotherapy and palliative surgery parallel recent pooled evidence. Contemporary series place median OS under systemic therapy in the 7- to 15-month range, consistent with our cohort’s outcomes. Meta-analytic data from 2024 suggest that palliative gastrectomy may extend survival in selected patients, concordant with our observed protective association; however, selection effects remain a concern, and randomized or carefully matched designs are needed to define patient subsets most likely to benefit.^[9,19]^

These data support a pragmatic, layered approach to prognostication and management in elderly GCPM. First, classify intraperitoneal disease by peritoneal implantation stage and ascites grade at baseline; these 2 variables together provide strong risk discrimination and are readily obtainable from routine exploration or imaging. Second, incorporate host metrics – ECOG, albumin, and NLR – into treatment decisions to appraise tolerance and benefit potential. Third, prioritize systemic chemotherapy in eligible patients and consider palliative surgery in carefully selected cases after multidisciplinary review. Embedding these elements into a standardized pathway may improve treatment allocation, goals-of-care discussions, and trial stratification. This study has several strengths. It focuses specifically on elderly GCPM, an underrepresented subgroup with distinct therapeutic constraints. It operationalizes 2 clinically accessible measures of peritoneal disease – peritoneal implantation stage and ascites grade – allowing direct translation into everyday practice without specialized tools. The analysis integrates host-related biomarkers (albumin and NLR) and treatment variables in a single multivariable framework, enabling simultaneous appraisal of disease burden, host status, and therapeutic exposure.

Treatment selection was not randomized and was strongly influenced by patients’ performance status, tumor burden, and overall clinical condition. Therefore, the observed survival advantages associated with systemic chemotherapy and palliative surgery should be interpreted as adjusted associations rather than causal effects. Selection bias and indication bias cannot be completely excluded despite multivariate adjustment.

Limitations warrant consideration. The retrospective, single-center design introduces potential selection bias and limits causal inference for treatment effects. Although we adjusted for multiple confounders, residual confounding is likely, particularly for the associations with chemotherapy and palliative surgery. Ascites grading and peritoneal staging were derived from routine imaging and operative reports; inter-observer variability may exist despite standardized criteria. Inflammatory and nutritional markers were assessed at baseline; dynamic changes during therapy were not modeled and could provide incremental prognostic value. We did not include detailed molecular features (e.g., human epidermal growth factor receptor 2, claudin 18.2, microsatellite instability, and Epstein-Barr virus), which are increasingly relevant for therapy selection and outcomes. Finally, external validation in independent multi-institutional cohorts was not performed. Future prospective multicenter studies are needed to confirm the robustness and clinical applicability of our findings. In addition, treatment selection bias deserves further consideration. Patients who received systemic chemotherapy or palliative surgery generally had better performance status, less extensive peritoneal disease, and relatively preserved nutritional status. These baseline advantages may partly explain their improved survival outcomes. Although multivariate adjustment was performed, residual confounding cannot be completely excluded in retrospective analyses. Prospective studies or propensity-matched analyses are needed to more definitively evaluate treatment effects in this population.

## 5. Conclusion

In elderly patients with GCPM, advanced peritoneal implantation stage, high ascites grade, poor performance status, hypoalbuminemia, and elevated NLR independently predict poor survival, whereas systemic chemotherapy and palliative surgery improve outcomes. These findings highlight the combined prognostic value of disease burden and host condition in guiding individualized management.

## Author contributions

**Conceptualization:** Airu Su, Ran Guo, Chao Wu.

**Funding acquisition:** Airu Su, Chao Wu.

**Data curation:** Airu Su, Ran Guo, Chao Wu.

**Formal analysis:** Airu Su, Ran Guo, Chao Wu.

**Investigation:** Chao Wu.

**Writing – original draft:** Airu Su, Ran Guo, Chao Wu.

**Writing – review & editing:** Airu Su, Ran Guo, Chao Wu.

## References

[1] Guchelaar NAD de Neijs MJ Noordman BJ. The prognostic value of peritoneal metastases in patients with gastric cancer: a nationwide population-based study. EClinicalMedicine. 2025;81:103109. 40026831 10.1016/j.eclinm.2025.103109 PMC11872448

[2] Guchelaar NAD Noordman BJ Welten MW. Systemic treatment strategies and outcomes of patients with synchronous peritoneal metastases of gastric origin: a nationwide population-based study. J Natl Compr Canc Netw. 2024;22:405–12. 39074509 10.6004/jnccn.2024.7013

[3] Kim HI Badgwell BD. Peritoneal oligometastasis in gastric cancer: diagnostic strategies, patient selection, and emerging therapeutic approaches. J Gastric Cancer. 2025;25:409–23. 40631471 10.5230/jgc.2025.25.e36 PMC12260789

[4] Lewis KA Diggs LP Badgwell BD. Educational review: updates on therapeutic strategies for gastric cancer with peritoneal metastasis. Ann Surg Oncol. 2025;32:3672–87. 40016614 10.1245/s10434-025-17069-3 PMC12268850

[5] de Moraes FCA Rego L Matheus G. Does malignant ascites define prognosis in gastric cancer with peritoneal spread? A systematic review and meta-analysis. J Gastrointest Cancer. 2025;56:206. 41099972 10.1007/s12029-025-01332-7

[6] Karalis JD Ju MR Feig R. Intensifying supportive care is associated with improved survival in gastric cancer patients with malignant ascites. J Surg Oncol. 2024;129:718–27. 38063245 10.1002/jso.27556

[7] Mariani A Triantafyllou E Kepenekian V Zaanan A Glehen O Karoui M. Management of peritoneal gastric metastasis: an update. Eur J Surg Oncol. 2025;51:109731. 40106892 10.1016/j.ejso.2025.109731

[8] Ruff SM. Cytoreductive surgery and hyperthermic intraperitoneal chemotherapy for gastric cancer peritoneal metastases. Surg Oncol Clin N Am. 2025;34:241–51. 40015802 10.1016/j.soc.2024.12.009

[9] Cho M Kim HS Jung M Hyung WJ. Perioperative intraperitoneal plus systemic chemotherapy and cytoreductive surgery with hyperthermic intraperitoneal chemotherapy for gastric cancer: phase Ib/II single-arm prospective study. J Gastrointest Surg. 2024;28:1095–103. 38705369 10.1016/j.gassur.2024.04.030

[10] Ji ZH Yu Y Liu G. Peritoneal cancer index (PCI) based patient selecting strategy for complete cytoreductive surgery plus hyperthermic intraperitoneal chemotherapy in gastric cancer with peritoneal metastasis: a single-center retrospective analysis of 125 patients. Eur J Surg Oncol. 2021;47:1411–9. 33293213 10.1016/j.ejso.2020.11.139

[11] Stefano M Perrina D Vallicelli C. Prophylaxis and treatment of peritoneal carcinomatosis of gastric origin using hyperthermic intraperitoneal chemotherapy: a systematic review and meta-analysis of randomized trials. J Gastrointest Surg. 2024;28:1185–93. 38599315 10.1016/j.gassur.2024.04.007

[12] Provenzano L Gwee YX Conca V. Unveiling the prognostic significance of malignant ascites in advanced gastrointestinal cancers: a marker of peritoneal carcinomatosis burden. Ther Adv Med Oncol. 2024;16:17588359241289517. 39502404 10.1177/17588359241289517 PMC11536604

[13] Fucà G Cohen R Lonardi S. Ascites and resistance to immune checkpoint inhibition in dMMR/MSI-H metastatic colorectal and gastric cancers. J Immunother Cancer. 2022;10:e004001. 35110358 10.1136/jitc-2021-004001 PMC8811606

[14] Wei ZH Tuo M Ye C. Prognostic value of neutrophil-to-lymphocyte ratio in gastric cancer patients undergoing neoadjuvant chemotherapy: a systematic review and meta-analysis. World J Gastrointest Oncol. 2024;16:4477–88. 39554738 10.4251/wjgo.v16.i11.4477 PMC11551644

[15] Tan S Zheng Q Zhang W Zhou M Xia C Feng W. Prognostic value of inflammatory markers NLR, PLR, and LMR in gastric cancer patients treated with immune checkpoint inhibitors: a meta-analysis and systematic review. Front Immunol. 2024;15:1408700. 39050856 10.3389/fimmu.2024.1408700 PMC11266030

[16] Manzanedo I Pereira F Pérez-Viejo E Serrano A. Gastric cancer with peritoneal metastases: current status and prospects for treatment. Cancers (Basel). 2023;15:1777. 36980663 10.3390/cancers15061777 PMC10046173

[17] Matsas S Aguiar PN Jr Del Giglio A. Neutrophil-to-lymphocyte ratio and platelet-to-lymphocyte ratio as biomarkers to prognosticate survival in advanced gastric cancer patients in the era of immunotherapy: a systematic review and meta-analysis. J Gastrointest Oncol. 2024;15:33–51. 38482212 10.21037/jgo-23-808 PMC10932683

[18] Deng M Qing Y Qiu D Sheng Y Zhou J Sun L. The prognostic value of pretreatment neutrophil-lymphocyte ratio and platelet-lymphocyte ratio in patients with esophageal cancer undergoing immunotherapy: a systematic review and meta-analysis. Front Oncol. 2025;15:1536920. 40027124 10.3389/fonc.2025.1536920 PMC11868166

[19] Yu P Ye Z Dai G. Neoadjuvant systemic and hyperthermic intraperitoneal chemotherapy combined with cytoreductive surgery for gastric cancer patients with limited peritoneal metastasis: a prospective cohort study. BMC Cancer. 2020;20:1108. 33198674 10.1186/s12885-020-07601-x PMC7667754

